# Editorial: Hurdling the Challenges of the 2019 IAAF World Championships

**DOI:** 10.3389/fspor.2019.00064

**Published:** 2019-12-03

**Authors:** Olivier Girard, Sebastien Racinais

**Affiliations:** ^1^Murdoch Applied Sports Science Laboratory, Murdoch University, Perth, WA, Australia; ^2^Research and Scientific Support, Aspetar Orthopaedic and Sports Medicine Hospital, Doha, Qatar

**Keywords:** athletics, heat stress, running mechanics, injury prevention, pacing strategies

The 17th International Association of Athletics Federations (IAAF) World Championships were staged in Doha, Qatar from September 27 to October 6, 2019. In recent years, the Qatari capital Doha had an extensive history as a host of international athletics events from the first ever IAAF Grand Prix in 1997 to the 2010 IAAF World Indoor Championships and the Doha Diamond League. However, the 2019 IAAF World Championships has been the largest sporting event held in Doha to date in terms of global reach and impact. Doha welcomed over 200 countries and 2,000 athletes with ~10,000 international guests, 30,000 spectators from outside Qatar, and more than 2,000 media personnel.

## Solving Real World Problems

This Research Topic includes athlete-centered research advancing the practical knowledge of coaches, exercise physiologists, sport biomechanists, sport analysts, sport physicians, and academic researchers. This unique collection of articles has direct practical applications to address specific challenges associated with 2019 IAAF World Championships and beyond. In particular, the papers address problems encountered by athletes during training or the actual competition, doping issues (Faiss et al.), and the merit of training interventions (i.e., altitude training; Slawinski et al.). The great diversity of athletic disciplines is reflected in this article collection spanning from 100-m sprint to marathon and walking events, and to pole vault. A total of 27 papers have been accepted including 18 original papers, 4 reviews, 4 perspectives, and 1 policy brief article. Those contributions feature over 110 authors arising from over 30 different research groups from more than 20 countries in Europe (e.g., France, Germany, Greece, Hungary, Italy, Netherlands, Spain, Switzerland, United Kingdom), Asia (e.g., Japan, Qatar, South Korea), Oceania (e.g., Australia, New Zealand), Africa (e.g., South Africa), North America (e.g., Canada, United States of America), and South America (e.g., Brazil).

This Research Topic represented a fantastic opportunity for the scientific community to provide up-to-date knowledge and propose solutions to real-world problems for elite competitors. Four key areas have been investigated:

1. Training and competing in hot environments
2. Management of the most common injuries and illnesses
3. Advanced biomechanical analyses
4. Racing and pacing.

## Training and Competing in Hot Environments

One of the unique characteristics of the 2019 IAAF World Championships was that it was hosted in the Middle-East for the first time. Nowadays, major sport competitions are no longer exclusively hosted in western countries. For example, despite existing since 1896, the summer Olympics were held in South America in 2016 for the first time, while so far have never been organized in Africa. Along the same line, the FIFA World cup exists since 1930 but was hosted for the first time in Asia in 2002, in Africa in 2010 and will come to Middle-East in 2022. This globalization of sport has brought several challenges to the athletes including travel fatigue, jetlag, and environmental conditions.

Hot and humid ambient conditions may play a major role during the endurance events of the 2019 IAAF World championships, the 2020 summer Olympics and many other major sport events. Heat stroke is the second highest cause of death in sport after cardiac conditions but should also be considered for spectators as it accounts for more deaths than all other natural disasters combined in the general population. Moreover, heat stress (due to a combination of heat and humidity) impacts exercise performance. Increasing muscle temperature benefits explosive activities, whereas high whole-body core temperature impairs prolonged exercise capacity in hot and humid environments. This Research Topic includes an original analysis of heat stroke prevalence in runners (Grundstein et al.) and provides applied recommendations for sport and medical communities (Racinais et al.), organizing committees (Bermon and Adami), and ultra-endurance athletes (Bouscaren et al.). It also includes papers addressing issues related to the development of new technologies (Muniz-Pardos et al.).

## Management of the Most Common Injuries and Illnesses

Beyond the specific issues of the environmental conditions, this Research Topic also presents applied recommendations to minimize injury and illness during major athletic championships (Edouard et al.). This is supported by original research on injury risk factor in athletics including the effect of team size (Edouard et al.) or a prospective epidemiological study (Carragher et al.). The specificity of the athletic disciplines is highlighted in a review on foot strength (Tourillon et al.) alongside original research on foot orthoses (Van Alsenoy et al.) and lower limb asymmetry (Girard et al.). There is also a specific focus on the 100 m (Fujita et al.) and pole vault (Edouard et al.) disciplines. Those eight articles related to potential injury risk factors represent the largest contingent of this Research Topic. This demonstrates the current interest of the research community in determining the risk factors and developing counter-measures.

## Advanced Biomechanical Analysis

The 100-m race is one the most popular athletics event that advanced biomechanical analysis has influenced over the years. Bezodis et al. analyzed the start and initial acceleration technique of the World's best male sprinters and hurdlers *in situ* during the finals of the 2018 IAAF World Indoor Championships and demonstrate many similarities between their kinematics and intersegment coordination profiles. From horizontal force-velocity-power profile data, Stavridis et al. also highlighted that high-level female sprinters are able to apply higher horizontally-oriented forces onto the ground during acceleration phase than the high-level female hurdlers. Using the longest force plate system in the World, Nagahara and Ohshima indicated how the location of the center of pressure on the starting block determines sprint start performance. Leg kinematic features of maximal speed sprinting at different leg length and step characteristics were further elucidated by the same research group using regression equations (Miyashiro et al.). Under treadmill running conditions, Moore et al. evaluated the effect of manipulating ground contact time on the metabolic cost of running in trained endurance runners. Biomechanical analysis not only is critical to improve tolerance to ground impact but also useful to ensure that athletes effectively comply with the rules in Race Walking. For the first time, Hanley et al. proposed an assessment of a large number of qualified IAAF racewalking judges from around the globe to accurately detect legal and non-legal technique, also providing flight times across a range of speeds to establish when athletes are likely to lose visible contact.

## Racing and Pacing

Pacing strategies have a considerable influence in determining race outcome by optimizing the limited energetic resources available. It is therefore crucial that young athletes, striving to reach the elite level, adequately develop their pacing behavior (Menting et al.). Middle distance races are amongst the most demanding athletic events, while these races are also characterized by a large variety of runner profiles (Sandford and Stellingwerff). Here, Hanley et al. questioned whether the draws for heats and lanes impact on placings and progression in 800-m championship racing, and proposed that the IAAF could consider allocating the inner lanes to faster athletes rather than the outer lanes. A detailed analysis of tactical behaviors that differentiate medallists in the 1,500-m race is also detailed by Sandford et al.. Knowledge pertaining to the underlying mechanisms and factors influencing the regulation of pace is still not well-understood. Here, Hettinga et al. explored whether World Championship and Olympic pacing profiles differ across middle-distance, long-distance and racewalk events for men and women, and also include for the first time data from the recently introduced 50 km women's racewalk event. Finally, Inoue et al. highlighted the impact of sex, performance level, and substantial speed reductions on pacing in a 24-h ultramarathon. This reinforces the need for future studies shedding more light on some of the factors influencing pacing strategies during official IAAF race formats.

## Moving Forward

IAAF Council awarded Eugene, USA, the 2021 IAAF World Championships. With the Doha meeting behind us, Eugene is already working with the IAAF to research innovative solutions for competition timing, scoring, measurement, and television production, using the latest technology. The IAAF is also determined to further accelerate the growth of women's athletics and Paralympics champions. This may in turn drive the development of new knowledge, using an integrative sports science approach, to improve performance of special athletic populations preparing major competitions (Tokyo 2020 Olympic and Paralympic games). On 12 October 2019, Eliud Kipchoge became the first human in history to break the 2-h marathon barrier (albeit in a non-official event). This would not have been possible without maximizing some of the factors—i.e., pacing strategy, running mechanics, and weather conditions—that formed the core of this Research Topic. As confirmed by this Research Topic, athletics continue to generate considerable interest globally. In this context, *Frontiers in Sport and Active Living* through its partnership with the Health and Science Department of the IAAF represents an ideal platform to disseminate new knowledge.

## Author Contributions

All authors listed have made a substantial, direct and intellectual contribution to the work, and approved it for publication.

### Conflict of Interest

The authors declare that the research was conducted in the absence of any commercial or financial relationships that could be construed as a potential conflict of interest.

